# The complete chloroplast genome sequence and phylogenetic analysis of *Viola grayi* (Violaceae)

**DOI:** 10.1080/23802359.2025.2566072

**Published:** 2025-11-19

**Authors:** Ah-reum Go, Yayoi Takahashi, Takaya Iwasaki, Ki-Oug Yoo

**Affiliations:** ^a^ Department of Biological Sciences, Kangwon National University, Chuncheon, South Korea; ^b^ Graduate School of Humanities and Sciences, Ochanomizu University, Tokyo, Japan

**Keywords:** Plastome, chloroplast genome, Violaceae, *Viola grayi*, phylogenetic analysis

## Abstract

*Viola grayi*, a caulescent species in subsection *Rostratae*, adapted to coastal dunes, with leathery leaves, is here reported with its first sequenced complete chloroplast genome (158,408 bp), comprising a large single-copy (86,662 bp), a small single-copy (17,994 bp), and pair of inverted repeats (26,876 bp each). Phylogenetic analysis of 22 *Viola* species, based on a 184,817 bp whole-plastome alignment, resolved the genus as monophyletic and placed *V. grayi* in a clade with *V. grypoceras* (BP = 100). This plastome provides a valuable genomic resource for future comparative, taxonomic, phylogenetic, and evolutionary studies.

## Introduction

**Figure 1.**
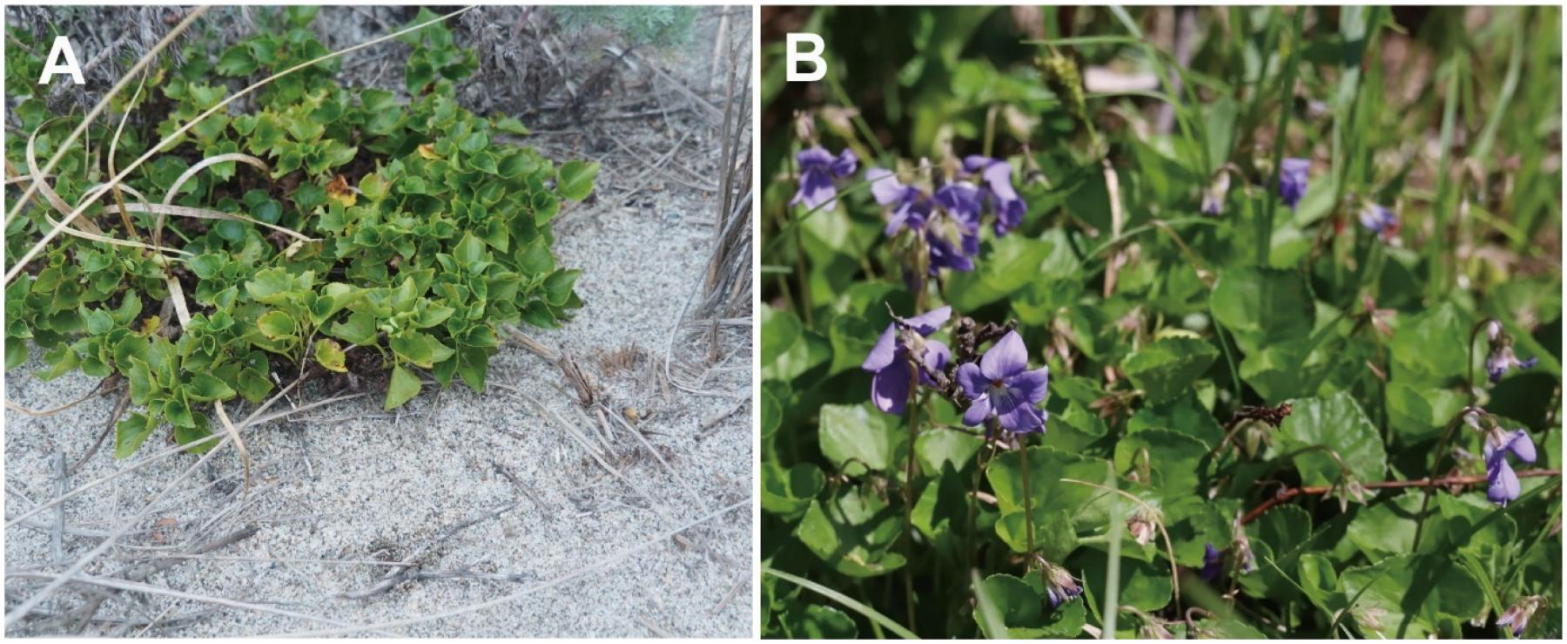
Photographic documentation of *Viola grayi*. (A) Habitat on coastal sand dunes (photo by Takaya Iwasaki) and (B) whole plant with thick glossy leaves, and five-lobed flowers with a blue-purple corolla and whitish base (photo by Kazuhiro Kaneko).

*Viola* L. is the largest genus within Violaceae, comprising approximately 658 species (Marcussen et al. 2022). *Viola grayi* Franch. et Sav. 1878 is a caulescent species endemic to Japan (Figure 1), originally described as glabrous with a short and thick spur (Franchet and Savatier 1879). The species primarily inhabits sandy shores from Tottori to Hokkaido, with additional occurrences in Aomori Prefecture. After its initial description, *V. grayi* was treated as a variety of *V. grypoceras* due to morphological similarity (Nakai 1925). However, the species inhabits coastal sand dunes and is characterized by thick slightly shiny leaves that differ from those of *V. grypoceras* and was reported as a distinct species (Nakai 1939). A recent study using SSR markers demonstrated that *V. grayi* is a concise group, distinct from closely related inland species based on SSR nuclear markers (Hirai et al. 2012). The findings are consistent with morphological classification and support its recognition as a distinct species.

According to Hirai et al. (2012), *V. grayi* exhibits low genetic diversity and high population differentiation, highlighting the need for its conservation. Additionally, research on germination traits (Kuroda and Sawada 2022) has revealed that extended periods of elevated winter temperatures prolong its seed dormancy and, indicating potential risk due to climate change. Furthermore, *V. grayi*, which is restricted to coastal sand dunes, requires specific conditions for seed germination. However, comprehensive genetic data, including complete chloroplast genomes, remain unavailable, limiting effective genetic conservation and accurate taxonomic resolution.

Chloroplast DNA is highly conserved and evolves more slowly, making it particularly useful for resolving phylogenetic relationships across various taxonomic levels (Palmer and Zamir 1982). Moreover, recent studies have suggested that complete chloroplast genome sequences can serve as super-barcodes for species identification in *Viola* (Cao et al. 2022). In such a context, we sequenced the complete chloroplast genome of *V. grayi* to determine its phylogenetic placement and establish a genomic reference for taxonomic and evolutionary investigations within the genus *Viola*. The contribution enhances the framework for resolving species boundaries and phylogenetic relationships, particularly within subsection *Rostratae*.

## Materials and methods

A *V. grayi* leaf sample was collected from a sandy beach in Tenbinno, Araya, Akita City, Akita Prefecture, Japan (39.711645N, 140.060217E) on 30 September 2023. The specimen was deposited at the Kangwon National University Herbarium (KWNU, https://biology.kangwon.ac.kr/biology; Ki-Oug Yoo, yooko@kangwon.ac.kr) under voucher number KWNU103412. The sample was rapidly dried in silica gel, and DNA was extracted using the DNA Plant Mini Kit (Qiagen Inc., Valencia, CA). The extracted genomic DNA was outsourced to LabGenomics Co., Ltd. (Seongnam, South Korea) for sequencing on an Illumina MiSeq platform (Illumina Inc., San Diego, CA), that generated 5,911,975 paired-end reads with an average read length of 301 bp. *De novo* assembly was conducted using Geneious 7.1.9 (Biomatters Ltd., Auckland, New Zealand). Sequencing depth was evaluated by mapping Illumina pair-end reads to the assembly using BWA v0.7.17, and per-base coverage was calculated using SAMtools v1.17 and visualized in Python 3.10. The complete chloroplast sequence was annotated using GeSeq (Tillich et al. 2017) and manually edited by comparison with the *V. acuminata* (GenBank accession number MW802528). Gene structures for cis and trans-splicing genes were generated using CPGview (http://www.1kmpg.cn/cpgview/; Liu et al. 2023). The annotation of the trans-spliced gene (*rps12*) was manually corrected by comparing it with the chloroplast genomes of three closely related species: *V. acuminata*, *V. grypoceras*, and *V. websteri* (GenBank accession numbers MW802528, OM055663, and MH229819, respectively). A circular genome map of the *V. grayi* chloroplast genome was created using OGDRAW version 1.3.1 (https://chlorobox.mpimp-golm.mpg.de/OGDraw.html/; Greiner et al. 2019). The phylogenetic position of *V. grayi* was inferred based on chloroplast genome sequences from 22 *Viola* species and an outgroup taxon, *Passiflora edulis* (family Passifloraceae: GenBank accession number NC_034285). Sequence alignment was performed using MAFFT v7.490 (Katoh and Standley 2013). Maximum-likelihood analysis was conducted using RAxML v8.2.12 (Stamatakis 2014) with the GTRGAMMA substitution model and 1000 bootstrap replicates. Taxonomy follows the intraspecific classification of Marcussen et al. (2022).

## Results

**Figure 2.**
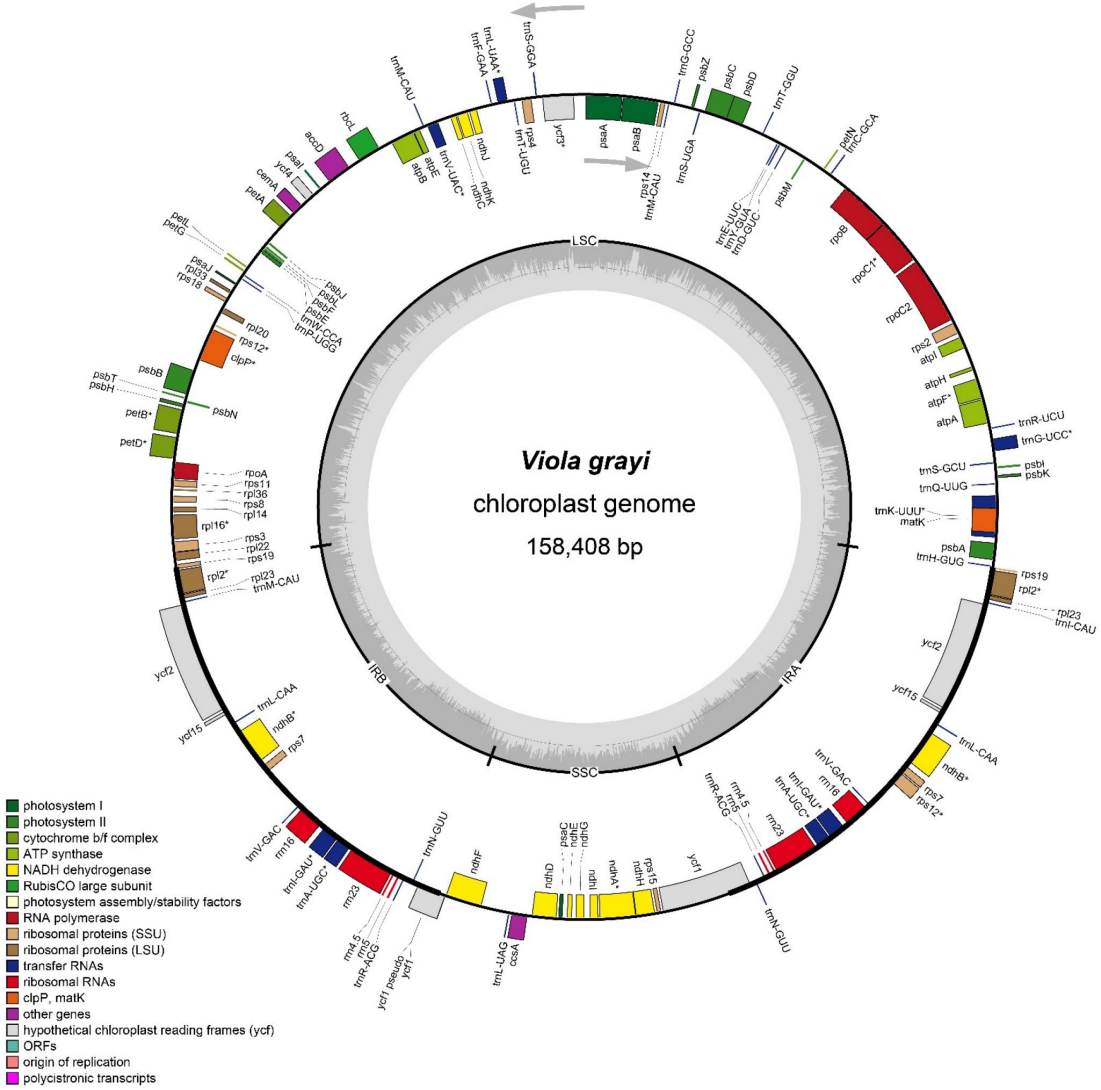
Circular map of the complete chloroplast genome of *Viola grayi*. The chloroplast quadripartite structure comprises a large single-copy (LSC) region, a small single-copy (SSC) region, and two inverted repeat (IRa and IRb) regions. Genes drawn outside the outer circle are transcribed clockwise, those inside are transcribed anticlockwise. The innermost gray histogram indicates the GC content across the genome.

The complete chloroplast genome of *V. grayi* (GenBank accession number: PV631333) is 158,408 bp in length with a GC content of 36.2%. Sequencing depth averaged 689×, with a minimum of 234× and a maximum of 3219× (Figure S1). The genome follows the typical quadripartite structure, comprising a large single-copy region of 86,662 bp, a small single-copy region of 17,994 bp, and two inverted repeats each 26,876 bp. In total, 131 genes, including 84 protein-coding genes, 37 tRNA genes, eight rRNA genes, and two pseudogenes (Figure 2).

Seventeen genes are duplicated within the IR regions, including seven protein-coding genes (*ndhB*, *rpl*2, *rpl*23, *rps*7, *rps12*, *ycf2*, and *ycf15*), four rRNA genes (*rrn16*, *rrn23*, *rrn4.5*, and *rrn5*), and six tRNA genes (*trnA-UGC*, *trnI-GAU*, *trnL-CAA*, *trnN-GUU*, *trnR-ACG*, and *trnV-GAC*). Nineteen intron-containing genes were identified, consisting of 13 protein-coding genes and six tRNA genes. Of these, 10 protein-coding genes (*atpF*, *ndhA*, *ndhB* [x2], *petB*, *petD*, *rpl2* [x2], *rpl16*, and *rpoC1*) and six tRNA genes (*trnA-UGC*, *trnG-UCC*, *trnI-GAU*, *trnK-UUU*, *trnL-UAA*, and *trnV-UAC*) contain a single intron. Among the protein-coding genes, three (*clpP*, *rps12*, and *ycf3*) contain two introns (Figure S2). The gene composition of the chloroplast genome of *V. grayi* is highly consistent with that of other previously reported *Viola* species.

**Figure 3.**
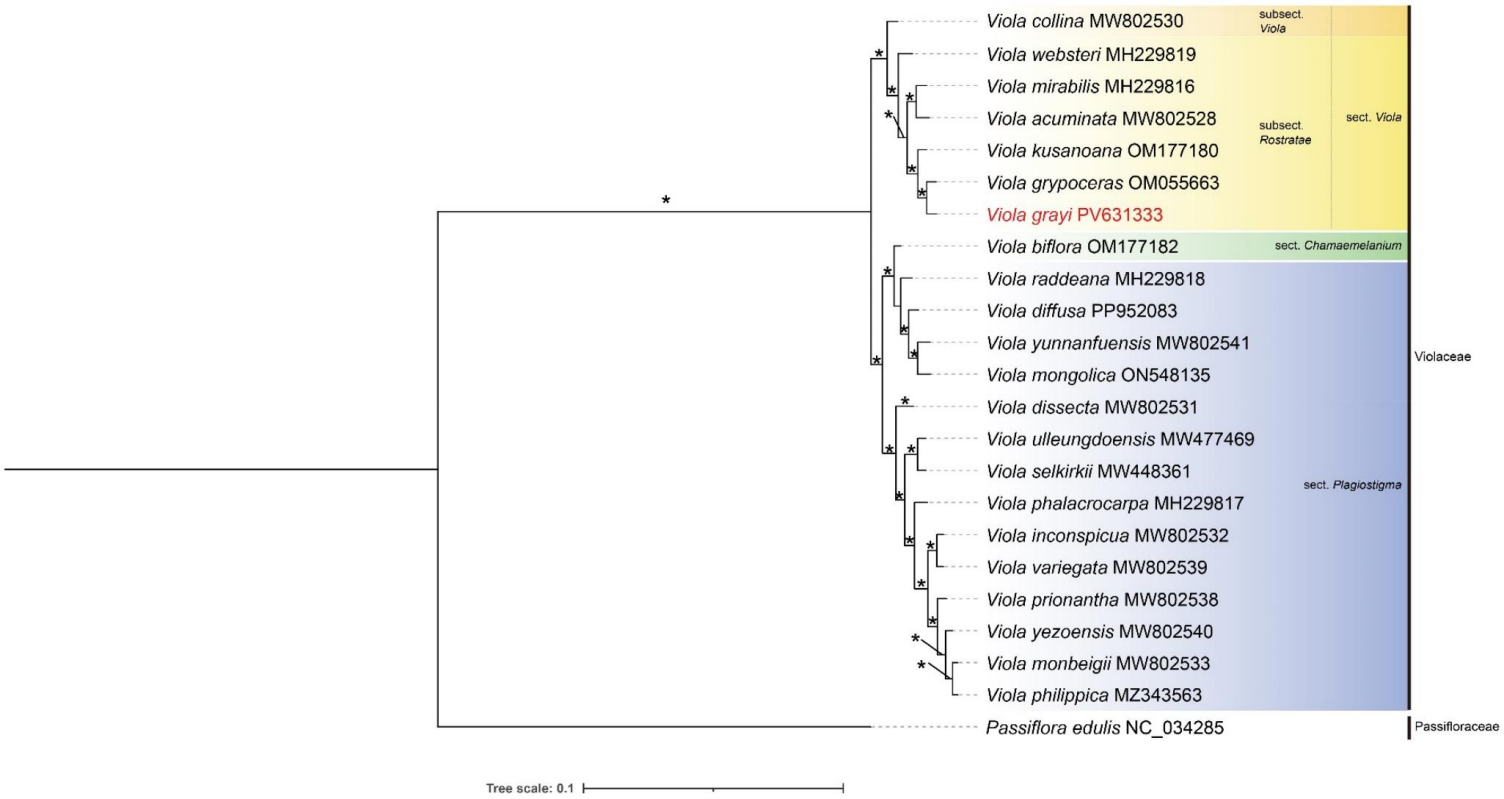
Maximum-likelihood (ML) tree inferred from an alignment of 184,817 bp of complete chloroplast genome sequences from 22 *Viola*, and one outgroup *Passiflora edulis* (NC_034285; Cauz-Santos et al. 2017). The position of *V. grayi* is highlighted in red. Bootstrap (BP) values are shown above the nodes, and an asterisk indicates nodes with 100% bootstrap support. The following sequences were used: *Viola acuminata* (MW802528; Cao et al. 2022), *V. biflora* (OM177182; Kang et al. 2024), *V. collina* (MW802530; Cao et al. 2022), *V. diffusa* (PP952083; Zhang et al. 2024), *V. dissecta* (MW802531; Cao et al. 2022), *V. grypoceras* (OM055663; Park et al. 2023), *V. inconspicua* (MW802532; Cao et al. 2022), *V. kusanoana* (OM177180; Kim et al. 2022; unpublished), *V. mirabilis* (MH229816; Cheon et al. 2019), *V. monbeigii* (Cao et al. 2022), *V. mongolica* (ON548135; Cao et al. 2022), *V. phalacrocarpa* (MH229817; Cheon et al. 2019), *V. philippica* (MZ343563; Cao et al. 2022), *V. prionantha* (MW802538; Cao et al. 2022), *V. raddeana* (MH229818; Cheon et al. 2019), *V. selkirkii* (MW448361; Go and Yoo 2023), *V. ulleungdoensis* (MW477469; Go and Yoo 2023), *V. variegata* (MW802539; Cao et al. 2022), *V. websteri* (MH229819; Cheon et al. 2019), *V. yezoensis* (MW802540; Cao et al. 2022), and *V. yunnanfuensis* W. Becker (MW802541; Cao et al. 2022).

For phylogenetic analysis, complete chloroplast genomes of 22 *Viola* species and one outgroup, including protein-coding, rRNA, and tRNA regions, were aligned, resulting in 184,817 bp. The results support the monophyly of *Viola* (Figure 3). Within the genus, two major clades were identified: one comprising sect. *Plagiostigma* and sect. *Chamaemelanium*, and the other consisting of sect. *Viola*. The sect. *Viola* clade was divided into two subclades, corresponding subsects. *Viola*, *Rostratae* as classified by Marcussen et al. (2022). *V. grayi* was placed within subsect. *Rostratae*, forming a strongly supported monophyletic clade with *V. grypoceras* (BP = 100).

## Discussion

This study reports the first complete chloroplast genome of *V. grayi*, which clarifies its phylogenetic position within the genus *Viola* and is essential for its conservation. *V. grayi* and *V. grypoceras*, historically confused due to morphological similarity, form a strongly supported monophyletic clade (BP = 100), confirming their close genetic relationship. Nucleotide diversity (Pi), calculated using DnaSP 6 (Rozas et al. 2017), was 0.00094, exceeding that of other closely related *Viola* species pairs (e.g. *V. monbeigii* and *V. philippica*, Pi = 0.00039). Although Pi values alone are insufficient for definitive species delimitation, they support the recognition of *V. grayi* as a distinct lineage. These genomic results are consistent with previous nuclear SSR marker studies demonstrating clear genetic differentiation (Hirai et al. 2012). The chloroplast genomic data presented here constitute a valuable resource for future taxonomic, phylogenetic, and evolutionary studies aimed at resolving species boundaries and elucidation of phylogenetic relationships within the genus *Viola.*

## Supplementary Material

resunmission_supplementary material.docx

## Data Availability

The genome sequence data that were obtained in this study are openly available in GenBank of NCBI (https://www.ncbi.nlm.nih.gov/) under the accession number PV631333. The associated BioProject, SRA, and Bio-Sample numbers are PRJNA 1261412, SRR 33514222, and SAMN 48425444, respectively.
