# Supplementary material for: The complete chloroplast genome sequence and phylogenetic analysis of Viola grayi (Violaceae)
Source: Mitochondrial DNA B Resour. 2025 Nov 19;10(12):1195–9. doi: 10.1080/23802359.2025.2566072 (PMC12632236; doi:10.1080/23802359.2025.2566072)
Supplement: resunmission_supplementary material.docx [file TMDN_A_2566072_SM2898.docx]

*
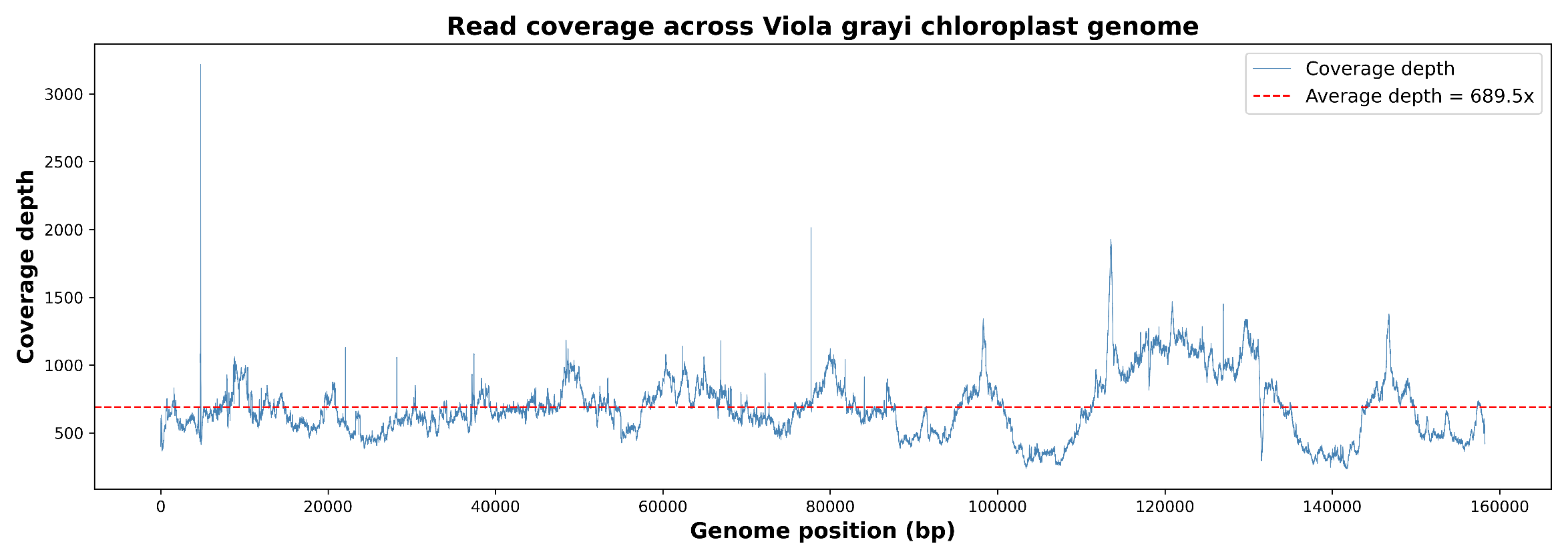
*

Figure S1. The paired-end reads generated an average coverage depth of 689×, ranging from 234× (minimum) to 3219× (maximum) across the chloroplast genome of *Viola grayi*.

**
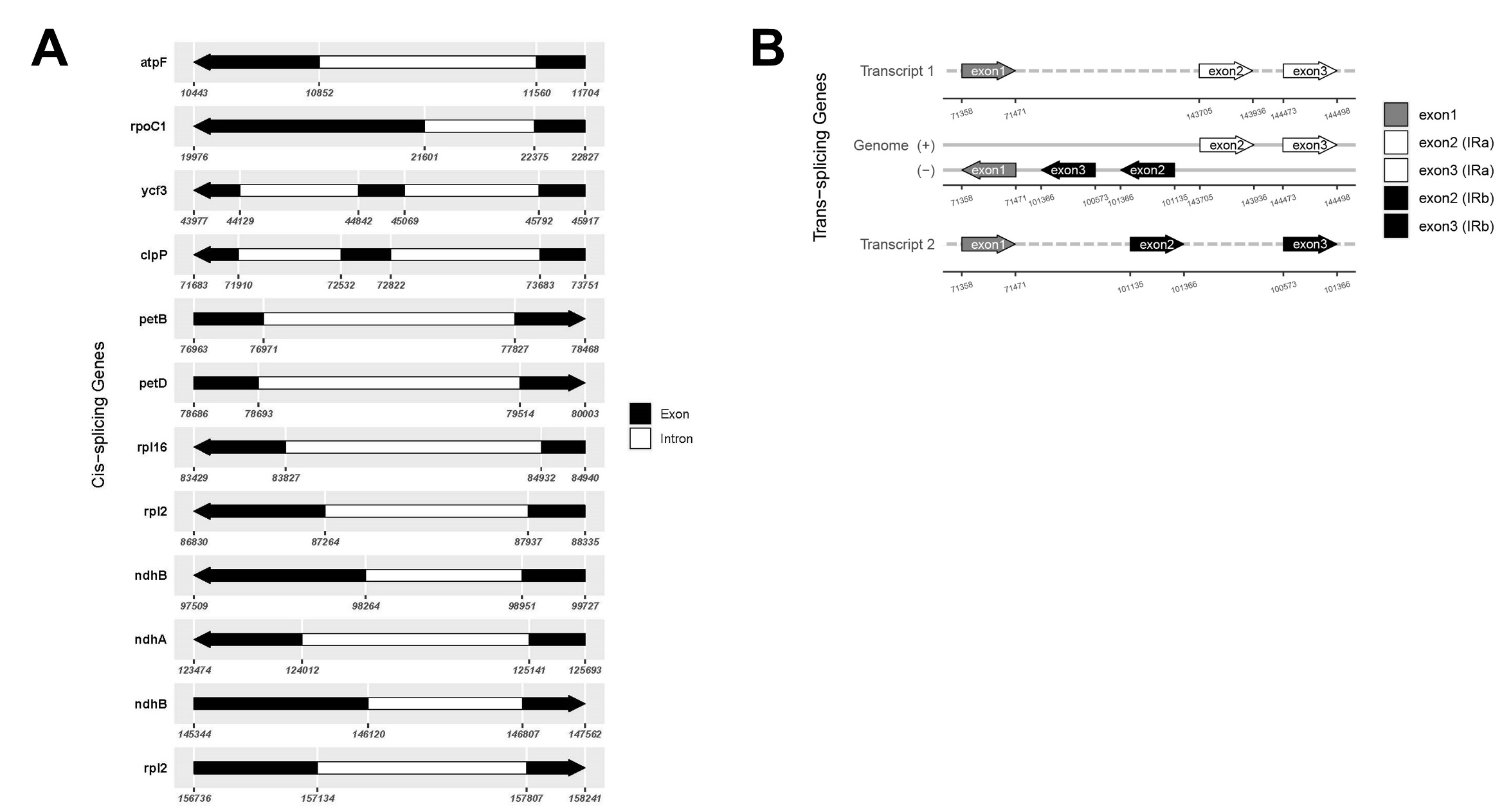
**

**Figure S2.** Schematic representation of the cis-spliced genes (A) and the trans-spliced *rps12* gene (B) in the chloroplast genome of *V. grayi*. White and black bars represent exons and introns, respectively, and arrows indicate gene orientation.
